# The Impact of Lifestyle Intervention Programs on Long-Term Cardiac Event-Free Survival in Patients With Established Coronary Artery Disease

**DOI:** 10.7759/cureus.76585

**Published:** 2024-12-29

**Authors:** Kimberly Kanemitsu, Baran Dilshad Hassan, Marika Mdivnishvili, Noor Abbas

**Affiliations:** 1 Clinical Sciences, Windsor University School of Medicine, Chicago, USA; 2 College of Medicine, Hawler Medical University, Erbil, IRQ; 3 Cardiac/Thoracic/Vascular Surgery, Jerarsi Hospital, Tbilisi, GEO; 4 Internal Medicine, Services Hospital Lahore, Lahore, PAK

**Keywords:** adherence, cardiac event-free survival, cardiac rehabilitation, coronary artery disease, lifestyle intervention, long-term outcomes, secondary prevention

## Abstract

Coronary artery disease (CAD) is a leading global cause of morbidity and mortality, necessitating comprehensive approaches for its management. This systematic review evaluates the long-term impact of structured lifestyle intervention programs on cardiac event-free survival in patients with established CAD. A total of eight studies, including randomized controlled trials (RCTs) and prospective cohort studies, were analyzed, encompassing diverse interventions such as cardiac rehabilitation, dietary modifications, exercise programs, and psychosocial support. The findings indicate that lifestyle interventions significantly improve event-free survival, reduce recurrent cardiac events, and enhance overall health markers as compared to usual care. Intensive interventions, such as comprehensive cardiac rehabilitation, showed the most pronounced benefits, including regression of coronary artery stenosis measured through angiographic imaging and a reduced need for revascularization (relative risk reduction up to 45%; p < 0.05). Flexible and accessible approaches, like home-based or telephonic rehabilitation, demonstrated potential in improving adherence, measured by program completion rates and self-reported lifestyle changes, and outcomes in specific populations such as elderly or high-risk patients. Limitations include variability in intervention intensity, small sample sizes in some studies, and differences in adherence definitions and measurement methods. This review highlights the critical role of lifestyle modifications as a cornerstone of secondary prevention strategies in CAD management and suggests that technology-based and demographic-specific interventions may hold promise for improving long-term outcomes. Future research should focus on long-term sustainability and optimizing tailored intervention designs.

## Introduction and background

Coronary artery disease (CAD) remains one of the leading causes of morbidity and mortality worldwide, significantly impacting patients’ quality of life and posing a substantial burden on healthcare systems [1]. CAD is often characterized by the progressive narrowing of coronary arteries due to atherosclerosis, leading to reduced blood flow, myocardial ischemia, and increased risk of adverse cardiac events such as myocardial infarction and stroke [2]. While pharmacologic therapies and surgical interventions, such as percutaneous coronary intervention (PCI) and coronary artery bypass graft (CABG) surgery, have effectively reduced mortality, they do not address underlying lifestyle factors that contribute to disease progression such as endothelial dysfunction, chronic inflammation, and metabolic imbalances. Consequently, there is a growing focus on lifestyle intervention programs as complementary approaches that address modifiable risk factors like poor diet, physical inactivity, and psychological stress to improve long-term outcomes in patients with established CAD [3]. Various studies suggest that structured lifestyle modification programs, including cardiac rehabilitation, dietary adjustments, physical activity regimens, and psychosocial support, may enhance event-free survival and improve prognosis among these patients.

Despite the existing evidence, there is a need to systematically evaluate the long-term impact of lifestyle intervention programs on cardiac event-free survival in patients with established CAD. Many prior studies have not adequately addressed long-term outcomes due to limited follow-up durations, small sample sizes, or methodological inconsistencies such as varying definitions of lifestyle adherence and differences in intervention intensity. These limitations make it challenging to draw robust conclusions about the sustained effects of lifestyle interventions on cardiac health. This systematic review aims to synthesize findings from high-quality studies on the effectiveness of lifestyle modification programs in prolonging cardiac event-free survival and reducing mortality among CAD patients. By focusing on studies with extended follow-up periods, this review intends to provide a comprehensive overview of how sustained lifestyle interventions contribute to improved cardiac outcomes, aiding clinicians in making informed recommendations for CAD management.

To structure this systematic review, a PICO (Population, Intervention, Comparison, Outcome) approach was adopted [4]. Population includes adult patients diagnosed with established coronary artery disease, encompassing both stable and unstable CAD, with no restrictions on prior interventions such as PCI or CABG. Intervention comprises various lifestyle modification programs, including but not limited to structured cardiac rehabilitation, exercise regimens, dietary changes, behavioral therapy, and mobile health-guided interventions. Mobile health-guided interventions typically involve the use of technology such as mobile apps, telemonitoring devices, or virtual coaching platforms to deliver personalized guidance, monitor progress, and improve adherence to lifestyle recommendations. The Comparison group, when present, includes patients receiving usual care without structured lifestyle interventions or those following standard follow-up routines. The primary Outcome of interest is long-term cardiac event-free survival, defined by the absence of major adverse cardiac events (MACE) such as myocardial infarction, stroke, or need for revascularization. Secondary outcomes include overall survival, reductions in recurrent cardiac events, improvements in physical fitness (measured by parameters such as peak oxygen uptake (Vo2peak) or exercise capacity tests), and adherence to lifestyle recommendations (evaluated through self-reported logs, adherence rates, or healthcare provider assessments).

The objective of this review is to evaluate and synthesize existing evidence on the impact of lifestyle intervention programs on long-term cardiac event-free survival in CAD patients. This review seeks to answer the following question: “In patients with established coronary artery disease, do structured lifestyle intervention programs, compared to usual care, significantly improve long-term cardiac event-free survival?” Subgroup analyses, such as those based on age, comorbidities, or intervention type (e.g., intensive rehabilitation vs. home-based programs), were also examined where data were available to identify variations in efficacy across different patient populations. By systematically examining data from relevant studies, this review aims to provide insights into the efficacy of lifestyle modifications as part of long-term management strategies in CAD, supporting clinicians and policymakers in promoting comprehensive, preventive care for improved patient outcomes. However, implementing lifestyle interventions at the population level presents challenges, including variability in healthcare access, resource allocation, patient adherence, and the need for tailored approaches to address diverse demographic and socioeconomic factors.

## Review

Materials and methods

Search Strategy

The search strategy for this systematic review was designed to capture a comprehensive range of studies that evaluate the impact of lifestyle intervention programs on long-term cardiac event-free survival in patients with established CAD. We began by selecting key databases known for their robust biomedical and clinical research resources, including PubMed, Cochrane Library, Embase, and Web of Science. Search terms were developed in accordance with the PICO framework, focusing on four core concepts: coronary artery disease, lifestyle intervention programs, comparison with usual care, and long-term cardiac outcomes. Boolean operators were employed to combine terms, ensuring inclusivity across synonyms and related keywords (e.g., “coronary artery disease” OR “CAD,” “lifestyle modification” OR “cardiac rehabilitation,” “event-free survival” OR “MACE”). Filters were applied to narrow the search to peer-reviewed studies with extended follow-up durations, randomized controlled trials, and cohort studies to maintain high-quality evidence. Following PRISMA (Preferred Reporting Items for Systematic Reviews and Meta-Analyses) guidelines [5], we documented each stage of the search and selection process to enhance transparency and reproducibility in study selection.

In addition to database searches, we conducted a manual review of reference lists from relevant articles to identify additional studies that met the inclusion criteria but may have been missed in the initial search. Gray literature, such as conference abstracts and unpublished studies, was considered to capture emerging evidence; however, only studies with substantial follow-up data and published statistical findings were included in the final review. The study selection process involved screening titles and abstracts for relevance, followed by full-text assessments. To ensure consistency and minimize bias, inter-reviewer reliability was assessed during screening, with two independent reviewers evaluating each study and resolving discrepancies through discussion or consultation with a third reviewer. Studies were included if they involved adult CAD patients and evaluated structured lifestyle interventions, such as diet, exercise, or stress management, as compared to usual care, with outcomes focusing on cardiac event-free survival and other secondary health markers. This rigorous approach ensured a comprehensive and representative analysis of lifestyle interventions on cardiac outcomes in CAD patients.

Eligibility Criteria

The eligibility criteria for this review were established to ensure the inclusion of high-quality studies that specifically address the impact of lifestyle intervention programs on long-term cardiac event-free survival in patients with established CAD. To be eligible, studies were required to focus on adult patients (18 years or older) diagnosed with CAD, without restriction on previous interventions such as percutaneous coronary intervention (PCI) or coronary artery bypass grafting (CABG). Only studies that evaluated structured lifestyle interventions were considered. Structured interventions were defined as those with clearly outlined components (e.g., standardized exercise regimens, dietary protocols, or behavior modification plans), a defined duration, and specified goals, typically involving healthcare professional oversight. Non-structured interventions, such as informal or self-guided lifestyle changes without predefined protocols, were excluded. Interventions had to be explicitly aimed at risk factor modification and adherence to a heart-healthy lifestyle. Studies that focused solely on pharmacologic treatments or surgical procedures without an integrated lifestyle component were excluded, as were those that did not provide detailed descriptions of the lifestyle interventions used.

In terms of study design, only RCTs, prospective cohort studies, and observational studies with control groups were eligible for inclusion, as these designs offer stronger evidence of causality between lifestyle interventions and cardiac outcomes. Qualitative or mixed-methods studies were excluded because the primary focus of this review was on quantitative evidence regarding cardiac event-free survival; however, these studies may provide valuable context for understanding adherence challenges, warranting separate exploration. Studies had to report on primary outcomes of long-term cardiac event-free survival, defined by the absence of major adverse cardiac events (MACE) such as myocardial infarction, stroke, or revascularization needs. Secondary outcomes, if reported, were also reviewed, including overall survival rates, recurrence of cardiac events, physical fitness improvements, and adherence to lifestyle modifications. Additionally, only studies with a minimum follow-up period of six months were included to ensure the assessment of sustained impact. Studies were limited to those published in English and available in the full-text format to allow thorough data extraction and analysis. The exclusion of non-English studies, while necessary due to resource limitations, may introduce language bias, potentially overlooking relevant evidence published in other languages. This limitation is acknowledged as a constraint in the review's scope.

Data Extraction

Data extraction was conducted systematically to ensure accuracy and consistency in capturing relevant details from each eligible study. Key information gathered included study characteristics (such as author, publication year, sample size, and country), patient demographics, specific components of the lifestyle intervention, and the nature of the comparison group. Outcomes related to cardiac event-free survival, including rates of MACE, overall mortality, and recurrent cardiac events, were meticulously recorded. Secondary outcomes, like improvements in physical fitness, adherence rates to lifestyle changes, and psychological markers, were also extracted where available. Statistical data, such as hazard ratios, relative risks, confidence intervals, and p-values, were collected to facilitate a quantitative synthesis of the results. Intervention fidelity, such as adherence rates to prescribed programs, was assessed where reported to evaluate the consistency and quality of implementation.

All extracted data were independently verified by two reviewers to minimize bias, with any discrepancies resolved through discussion and, when necessary, consultation with a third reviewer. The resolution process was qualitatively tracked, with disagreements documented and categorized by type (e.g., data interpretation or inclusion criteria). Software tools, including EndNote for reference management and Covidence for screening and data extraction, were employed to streamline the process and maintain organization. This rigorous approach to data extraction ensured that all relevant variables were consistently captured for comprehensive analysis.

Data Analysis and Synthesis

Data analysis and synthesis focused on identifying and summarizing patterns in the effects of lifestyle intervention programs on long-term cardiac event-free survival in patients with CAD. Quantitative data from each study, including hazard ratios, relative risks, and confidence intervals, were systematically reviewed to evaluate the impact of lifestyle interventions on primary outcomes such as the incidence of MACE and overall mortality. A descriptive synthesis approach was employed to integrate findings across studies, highlighting variations in intervention types, patient demographics, and follow-up durations. Heterogeneity among studies was assessed narratively, and subgroup analyses were conducted where data allowed, examining differences by patient demographics (e.g., age, comorbidities) or intervention types (e.g., intensive rehabilitation, home-based programs, or telephonic support). Qualitative findings, such as insights into patient adherence, psychosocial responses, and intervention implementation, were systematically integrated into the overall synthesis by categorizing themes across studies, comparing trends, and assessing their influence on primary and secondary outcomes. This comprehensive approach provided context for understanding the potential of lifestyle interventions to improve long-term outcomes in CAD patients, even without performing a formal meta-analysis.

Results

Study Selection Process

**Figure 1:**
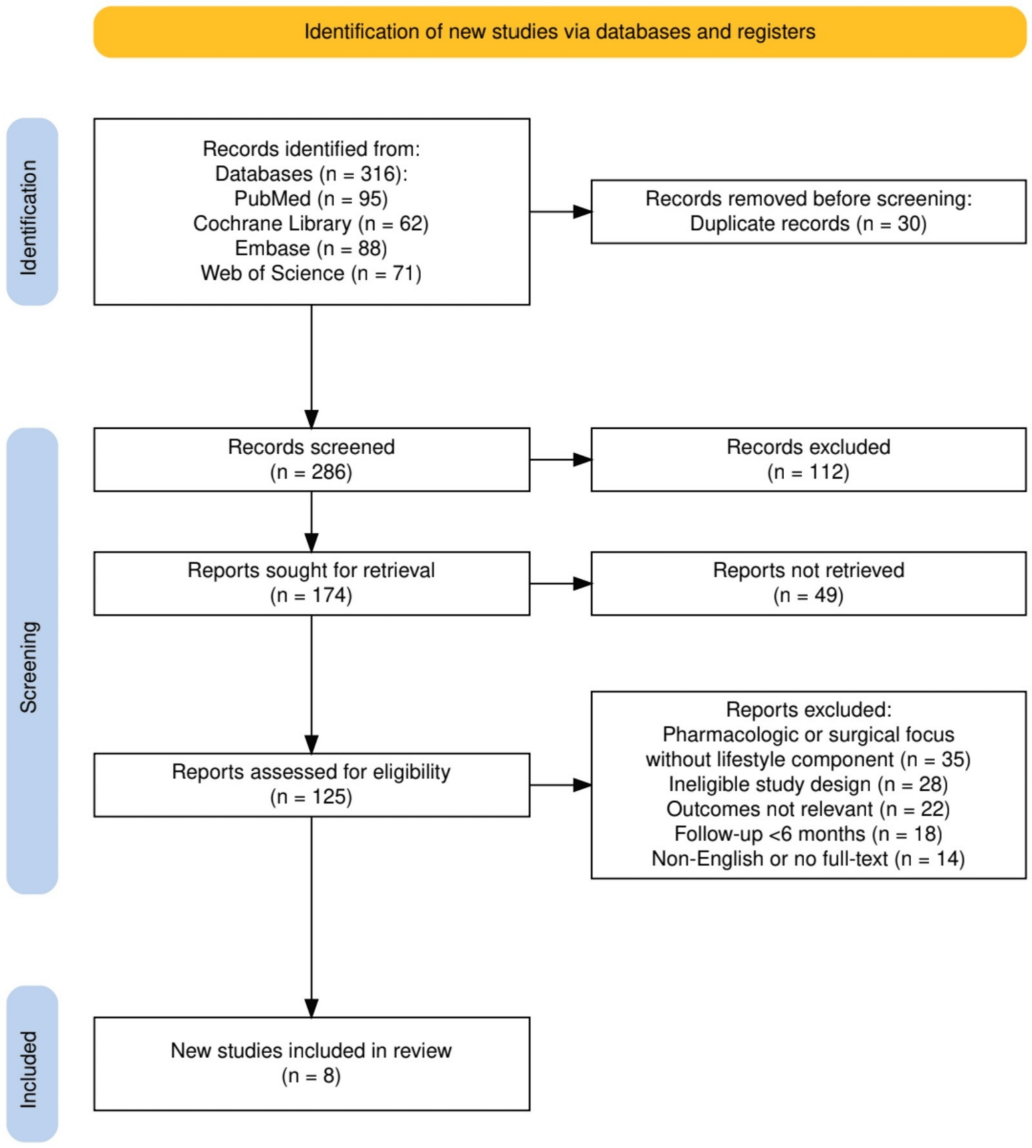
The PRISMA flowchart represents the study selection process PRISMA: Preferred Reporting Items for Systematic Reviews and Meta-Analyses

The study selection process followed the PRISMA (Preferred Reporting Items for Systematic Reviews and Meta-Analyses) guidelines. Initially, 316 records were identified across four databases: PubMed (95), Cochrane Library (62), Embase (88), and Web of Science (71). After removing 30 duplicate records, 286 studies were screened for relevance. Of these, 112 studies were excluded during the initial screening, and 49 reports could not be retrieved for further evaluation. The remaining 125 reports were assessed for eligibility, with 117 excluded based on predefined criteria (e.g., pharmacologic or surgical focus, ineligible study design, irrelevant outcomes, short follow-up periods, or non-English publications). Ultimately, eight studies meeting the inclusion criteria were included in the systematic review. Figure 1 represents the study selection process.

Characteristics of the Selected Studies

Table 1 provides an overview of the studies included in this systematic review, summarizing key details such as population characteristics, interventions, comparisons, outcomes, follow-up durations, and major findings. Each study explores the impact of lifestyle interventions on cardiac outcomes in patients with CAD. Interventions range from intensive rehabilitation programs to telephonic and mobile-based approaches, with outcomes measured over varying follow-up periods.

**Table 1:** Summary of studies included in the article CAD: Coronary Artery Disease; CABG: Coronary Artery Bypass Graft; CHD: Coronary Heart Disease; PTCA: Percutaneous Transluminal Coronary Angioplasty; PCI: Percutaneous Coronary Intervention; RR: Risk Ratio; HR: Hazard Ratio; MCR: Mobile Cardiac Rehabilitation; BMI: Body Mass Index; Vo2peak: Peak Oxygen Uptake

Study	Population (Sample Size)	Intervention	Comparison	Outcome Measures	Follow-Up Duration	Statistical Data	Key Findings	Concurrent Medications
Ornish et al., 1998 [6]	48 patients with moderate to severe CAD (35 completed 5-year follow-up)	Intensive lifestyle changes: 10% fat whole foods vegetarian diet, aerobic exercise, stress management, smoking cessation, group psychosocial support	Usual care with moderate lifestyle changes	Adherence to lifestyle changes, coronary artery diameter stenosis, cardiac events	5 years	- Risk ratio (RR) for any cardiac event: 2.47 (95% CI: 1.48-4.20) - Significant stenosis regression in the experimental group (P=.001)	Intensive lifestyle changes led to a 7.9% regression in coronary artery stenosis and reduced cardiac events (25 events vs. 45 in control). Control group showed 27.7% stenosis worsening and higher event rates.	Experimental group: No lipid-lowering drugs, 14 patients used aspirin. Control group: 9 (60%) used lipid-lowering drugs, 11 used aspirin.
Plüss et al., 2011 [7]	224 patients post-myocardial infarction or CABG	Expanded cardiac rehabilitation: stress management, increased physical training, 5-day stay at a 'patient hotel,' cooking sessions	Standard cardiac rehabilitation	Cardiovascular death, myocardial infarction, hospital readmissions, days in hospital for cardiovascular reasons	5 years	- Hazard ratio for cardiovascular events: 0.69 (P=0.049) - MI hazard ratio: 0.47 (P=0.047) - Median hospital days: 6 vs. 10 (P=0.02)	Expanded rehab reduced cardiovascular events (47.7% vs. 60.2%), myocardial infarction incidence (10.8% vs. 20.3%), and hospital days (6 vs. 10) compared to standard rehabilitation.	Concurrent standard cardiac medications as part of usual care.
Stewart et al., 2017 [8]	15,486 patients with stable CHD across 39 countries	Self-reported exercise (mild, moderate, vigorous) in metabolic equivalents of task hours/week	Sedentary to lower levels of physical activity	All-cause mortality, cardiovascular mortality, myocardial infarction, stroke	3.7 years (median)	- All-cause mortality HR: 0.90 (95% CI: 0.87-0.93, adjusted) - Cardiovascular mortality HR: 0.92 (95% CI: 0.88-0.96)	Increased physical activity associated with lower mortality in stable CHD patients, especially in higher-risk groups. Largest mortality reduction observed between sedentary patients and those with modest activity levels.	Standard pharmacological therapies (statins, antiplatelets, ACE inhibitors, beta-blockers) were part of routine care; the study adjusted for these in the multivariate analysis.
Wallner et al., 1999 [9]	60 patients with CAD post-PTCA	Intensified lifestyle intervention (diet, physical activity, body composition management)	Conventional treatment by cardiologists and general practitioners	Need for further revascularization, lifestyle-related measures	26 months	- Event-free survival probability: 0.89 (intervention) vs. 0.57 (control), P=0.0055 - Relative risk of revascularization: 0.26 (95% CI: 0.09–0.74)	Intensified lifestyle intervention significantly reduced revascularization needs (3/28 vs. 14/32) and improved event-free survival in CAD patients post-angioplasty compared to conventional care.	Standard CAD medications were part of routine care for both groups, including antiplatelets, beta-blockers, and statins.
Lisspers et al., 2005 [10]	88 patients post-PCI	Behaviorally oriented cardiac rehab with aggressive lifestyle changes (smoking cessation, diet, exercise, stress)	Standard care	Recurrent coronary events, cardiovascular mortality	5 years	- Coronary event rate: 30.4% (intervention) vs. 53.7% (control) - Cardiovascular mortality: 2.2% (intervention) vs. 14.6% (control)	Significant reduction in coronary events and cardiovascular mortality with aggressive lifestyle changes post-PCI, highlighting the long-term benefits of lifestyle-oriented secondary prevention.	Standard post-PCI pharmacological therapies (e.g., antiplatelets, beta-blockers, statins) were part of routine care.
Snoek et al., 2021 [11]	179 elderly CAD patients (65+ years) in Europe	6-month home-based mobile cardiac rehabilitation (telemonitoring, motivational coaching)	No cardiac rehabilitation	Peak oxygen uptake (Vo2peak), adverse events	6 and 12 months	- Vo2peak improvement at 6 months: +1.2 (95% CI, 0.2 to 2.1) mL/kg/min - Vo2peak improvement at 12 months: +0.9 (95% CI, 0.05 to 1.8) mL/kg/min	Home-based MCR significantly improved physical capacity in elderly CAD patients who declined clinic-based rehab. Safe with low adverse event incidence, showing promise as an alternative for nonparticipants in conventional rehab.	Both groups received standard care for coronary artery disease, including guideline-recommended pharmacological therapies such as antiplatelets, beta-blockers, and statins. No differences in medication use between groups were reported.
Leemrijse et al., 2016 [12]	374 patients with recent coronary events (<8 weeks post-hospitalization for AMI or (un)stable angina pectoris)	'Hartcoach' telephonic lifestyle intervention every 4 weeks for 6 months, in addition to usual care	Usual care only	BMI, waist circumference, physical activity, diet, self-management, anxiety, coronary risk scores	6 months	- BMI: b = -0.32 (95% CI: -0.63 to -0.003) - Waist circumference: b = -1.71 (95% CI: -2.73 to -0.70) - Physical activity: b = 15.08 (95% CI: 0.13 to 30.04)	'Hartcoach' modestly improved coronary risk factors, including BMI, physical activity, diet, and reduced anxiety, indicating its potential as a maintenance program for lifestyle changes in CAD patients.	Both groups received usual care, including guideline-recommended pharmacological therapies such as antiplatelets, beta-blockers, statins, and ACE inhibitors/ARBs. No differences in medication use between groups were reported.
Madssen et al., 2014 [13]	49 CAD patients post-cardiac rehabilitation	12-month maintenance program: monthly supervised high-intensity interval training (HIIT), written exercise program, quarterly exercise testing	Usual care	Peak oxygen uptake (Vo2peak), physical activity level, quality of life, blood biomarkers	12 months	- Vo2peak change: Intervention group 27.9 to 28.8 mL/kg/min, Control group 32.0 to 32.8 mL/kg/min (P=0.22)	No significant improvement in exercise adherence or Vo2peak compared to usual care. Suggests infrequent supervised sessions may be inadequate for sustained exercise capacity improvement in CAD patients.	All participants were on optimal medical therapy, including beta-blockers, antiplatelets, statins, and ACE inhibitors/ARBs. No changes in medication use occurred during the study.

Quality Assessment

To ensure the reliability and validity of findings in this systematic review, a quality assessment of included studies was conducted using the Cochrane Risk of Bias Tool for randomized controlled trials and the Newcastle-Ottawa Scale (NOS) for observational studies. Studies were rated as "High," "Moderate to High," or "Moderate," reflecting their adherence to methodological standards and potential biases. "High" quality indicated minimal bias and robust methodologies while "Moderate" quality highlighted significant limitations, such as small sample sizes or reliance on self-reported measures, which may affect the reliability or generalizability of findings. "Moderate to High" indicated generally reliable studies with minor limitations. These ratings, summarized in Table 2, were factored into the overall synthesis to account for variability in study rigor.

**Table 2:** Quality assessment of included studies using appropriate appraisal tools

Study	Quality Assessment Tool Used	Quality Assessment Summary	Overall Quality
Ornish et al., 1998 [6]	Cochrane Risk of Bias Tool	- Randomization: Low risk due to clearly described randomization. - Blinding: High risk as blinding of participants and researchers was not possible. - Incomplete Outcome Data: High risk due to 27% attrition (35/48 completed). - Other Biases: Low risk as intervention and control groups were well-matched.	Moderate
Plüss et al., 2011 [7]	Cochrane Risk of Bias Tool	- Randomization: Low risk due to appropriate allocation methods. - Blinding: Moderate risk; outcome assessors were blinded but not participants. - Incomplete Outcome Data: Low risk with detailed follow-up. - Selective Reporting: Low risk; all outcomes specified in the protocol were reported.	High
Stewart et al., 2017 [8]	Newcastle-Ottawa Scale (NOS)	- Selection: Low risk due to a large, representative population and clearly defined exposure. - Comparability: Moderate risk as self-reported physical activity may introduce bias. - Outcome Assessment: Low risk with standardized mortality data collection.	High
Wallner et al., 1999 [9]	Cochrane Risk of Bias Tool	- Randomization: Low risk due to clearly described randomization. - Blinding: Moderate risk as blinding of participants was not feasible. - Incomplete Outcome Data: Low risk; attrition was minimal and well-documented. - Other Biases: Moderate risk due to reliance on self-reported adherence.	Moderate
Lisspers et al., 2005 [10]	Cochrane Risk of Bias Tool	- Randomization: Low risk with appropriate allocation concealment. - Blinding: High risk due to the nature of behavioral interventions. - Incomplete Outcome Data: Moderate risk as attrition was higher in the control group. - Selective Reporting: Low risk with comprehensive reporting of all outcomes.	Moderate
Snoek et al., 2021 [11]	Cochrane Risk of Bias Tool	- Randomization: Low risk as randomized allocation was clearly defined. - Blinding: Moderate risk due to lack of participant blinding. - Incomplete Outcome Data: Low risk with detailed follow-up across intervention and control groups. - Selective Reporting: Low risk as all planned outcomes were reported.	Moderate to High
Leemrijse et al., 2016 [12]	Newcastle-Ottawa Scale (NOS)	- Selection: Low risk with a clear definition of population and intervention. - Comparability: Moderate risk as allocation to intervention was not randomized. - Outcome Assessment: Low risk with reliable measures for BMI, physical activity, and anxiety.	Moderate
Madssen et al., 2014 [13]	Cochrane Risk of Bias Tool	- Randomization: Low risk with clear allocation methods. - Blinding: High risk as participants were aware of their intervention group. - Incomplete Outcome Data: Moderate risk with incomplete adherence reporting. - Other Biases: Moderate risk due to small sample size, limiting generalizability.	Moderate

Key biases included attrition bias from high dropout rates, potentially skewing intervention effects, and self-reported measures, which could introduce recall or social desirability bias. Other biases, such as small sample sizes or limited generalizability to broader CAD populations, were noted in studies rated as "Moderate." Despite these limitations, most studies provided valuable insights into the impact of lifestyle interventions, underscoring the importance of cautious interpretation when drawing conclusions from studies with lower quality ratings.

Discussion

This systematic review highlights that lifestyle intervention programs have a considerable impact on improving long-term cardiac event-free survival in patients with coronary artery disease (CAD). Intensive lifestyle modification programs, such as those examined by Ornish et al. [6], demonstrated a 7.9% regression in coronary artery stenosis and a significant reduction in cardiac events compared to usual care, with a risk ratio of 2.47 (95% CI: 1.48-4.20). This implies that patients with obstructive CAD, including those requiring PCI, were included in the study population. Similarly, Plüss et al. reported that expanded cardiac rehabilitation reduced cardiovascular events and myocardial infarction incidence among patients post-myocardial infarction or CABG, with hazard ratios of 0.69 (P=0.049) and 0.47 (P=0.047), respectively, confirming that patients with bypass grafts were also part of the analyzed cohorts [7]. These findings emphasize the potential of intensive and structured lifestyle changes, including dietary adjustments, physical training, and psychosocial support, to mitigate the progression of CAD and reduce adverse cardiac outcomes over an extended period.

Additionally, studies focusing on more specific intervention components, such as physical activity and telephonic support, provided further insights into the effectiveness of these lifestyle programs. Stewart et al. demonstrated that increased physical activity was associated with a reduction in all-cause mortality (HR: 0.90, 95% CI: 0.87-0.93) and cardiovascular mortality (HR: 0.92, 95% CI: 0.88-0.96) among stable coronary heart disease patients, particularly those with previously sedentary lifestyles [8]. In another example, the Hartcoach telephonic intervention by Leemrijse et al. showed moderate improvements in BMI (b = -0.32, 95% CI: -0.63 to -0.003) and waist circumference, suggesting a modest but meaningful effect on physical risk factors [12]. Notably, Snoek et al. observed that a mobile cardiac rehabilitation program significantly improved Vo2peak in elderly patients over six months, further supporting the utility of accessible and flexible rehabilitation options [11]. Overall, these studies collectively demonstrate that both intensive and moderate lifestyle interventions can positively impact long-term outcomes in CAD patients, with specific benefits depending on the intervention intensity, structure, and patient demographics.

A critical aspect of the included studies is the consistent use of standard pharmacological therapies across intervention and control groups, which strengthens the validity of the observed lifestyle intervention outcomes. For instance, Ornish et al. demonstrated significant regression in coronary artery stenosis and reduced cardiac events despite the absence of lipid-lowering drugs in most experimental group patients, highlighting the standalone efficacy of intensive lifestyle changes [6]. Similarly, Plüss et al. and Stewart et al. accounted for concurrent medication use, ensuring that improvements in cardiovascular outcomes were attributed to the interventions rather than pharmacological confounders [7,8]. This pattern was also evident in studies such as by Wallner et al. and Snoek et al., where standardized medication regimens provided a stable background, allowing lifestyle modifications to emerge as the primary driver of improved cardiac outcomes [9,11]. These findings underscore the complementary role of lifestyle interventions alongside optimal medical therapy, emphasizing their value in enhancing event-free survival and physical fitness in CAD patients.

Additionally, the diverse delivery methods of these lifestyle programs, ranging from intensive rehabilitation to mobile and telephonic interventions, highlight their adaptability across different patient populations and settings. Programs like Hartcoach [12] and the mobile cardiac rehabilitation studied by Snoek et al. demonstrated significant benefits, such as improved physical activity levels, reduced BMI, and enhanced peak oxygen uptake, even in elderly patients or those unwilling to participate in conventional programs [11]. However, findings from Madssen et al. suggest that infrequent supervised sessions may be insufficient for sustained improvements, reinforcing the need for tailored approaches based on intervention intensity and patient demographics [13]. Together, these studies illustrate the broad potential of structured lifestyle programs to complement pharmacological care, adapt to patient needs, and provide scalable solutions for CAD management, particularly in underserved populations or those with limited access to traditional rehabilitation facilities.

The findings of this systematic review align with existing literature that underscores the efficacy of lifestyle interventions in managing CAD. For instance, a meta-analysis by Janssen et al. [14] demonstrated that lifestyle modification programs significantly reduced all-cause mortality and cardiac readmissions in CAD patients, corroborating the positive outcomes observed in studies like those by Ornish et al. [6] and Plüss et al. [7]. Similarly, a systematic review by de Waure et al. found that multifactorial lifestyle interventions led to an 18% reduction in fatal cardiovascular events among patients with established coronary heart disease, supporting the benefits of comprehensive lifestyle changes [15]. These consistencies reinforce the notion that structured lifestyle programs are integral to secondary prevention strategies in CAD management.

However, some discrepancies exist in the literature regarding the long-term sustainability of these interventions. A meta-analysis by Bergum et al. (2021) indicated that while lifestyle interventions resulted in modest reductions in systolic blood pressure, the effects on total cholesterol were negligible after 24 months [16]. This contrasts with the significant improvements in physical fitness and reductions in cardiac events reported in studies like Stewart et al. [8] and Snoek et al. [11]. These differences may stem from variations in intervention intensity, patient adherence, and follow-up durations across studies. Notably, this review contributes new insights by highlighting the effectiveness of home-based and telephonic interventions, such as those by Leemrijse et al. [12] and Snoek et al. [11], in improving cardiac outcomes. These findings suggest that flexible, patient-centered approaches can enhance adherence and extend the benefits of lifestyle modifications to a broader patient population, addressing gaps identified in previous research regarding the accessibility and sustainability of traditional rehabilitation programs.

The findings of this review highlight the critical role of lifestyle interventions in enhancing outcomes for patients with coronary artery disease (CAD), advocating for their integration into comprehensive CAD management strategies. Structured lifestyle programs, ranging from intensive cardiac rehabilitation and dietary modifications to accessible home-based and telephonic interventions, can significantly improve cardiac event-free survival, reduce recurrent events, and support overall health [6,12,17]. For instance, Ornish et al. [6] demonstrated regression of coronary stenosis while Leemrijse et al. [12] showed improved adherence and reduced BMI through telephonic coaching, emphasizing the importance of patient education. Tailored interventions addressing demographic and clinical needs, such as telephonic coaching with shorter, frequent calls for elderly patients or app-based solutions for younger individuals, further optimize outcomes [12,18].

Barriers to implementation, including resource constraints and patient resistance, can be mitigated through scalable solutions like group education sessions, mobile health tools, and community-based initiatives [17,18]. Financial incentives or subsidies could improve accessibility in underserved populations while regular follow-up and assessments are essential to sustaining adherence and maximizing long-term benefits [18,19]. By integrating such personalized and scalable approaches, clinicians can enhance survival, reduce healthcare burdens, and improve the quality of life of CAD patients [19].

Lifestyle interventions improve cardiac event-free survival in CAD patients through several interconnected biological, behavioral, and psychosocial mechanisms [20]. Biologically, lifestyle changes such as dietary adjustments, regular physical activity, and stress management reduce risk factors like high blood pressure, cholesterol levels, and systemic inflammation, each of which directly impacts the progression of atherosclerosis and coronary artery disease [21]. For instance, aerobic exercise and a heart-healthy diet can lower LDL cholesterol, increase HDL cholesterol, and reduce inflammation markers such as C-reactive protein (CRP), leading to improved endothelial function and decreased plaque formation. Studies have shown that CRP levels can decrease by up to 30% with regular exercise and dietary changes, directly lowering the risk of adverse cardiac events [21]. Behaviorally, structured programs encourage sustainable health habits, helping patients adhere to medication regimens, adopt consistent exercise routines, and make informed dietary choices, which collectively contribute to reduced rehospitalization rates and improved quality of life.

Psychosocially, interventions that incorporate stress management, counseling, or support groups address mental health factors like anxiety and depression, which are known to exacerbate cardiac risk and affect lifestyle adherence [22]. For example, participation in stress management programs has been associated with a 20% reduction in recurrent cardiac events by improving coping mechanisms and reducing sympathetic nervous system activation. These mechanisms interact synergistically; for instance, reducing psychological stress through counseling improves behavioral adherence while better adherence to exercise regimens enhances biological outcomes, such as lipid profile improvements and blood pressure control. Together, these pathways translate into tangible clinical benefits, including reductions in hospitalization rates, mortality, and MACE. This comprehensive integration of physiological, behavioral, and psychosocial approaches underscores the effectiveness of lifestyle interventions in reducing cardiac events and enhancing long-term survival in CAD patients.

This review has several strengths, including a rigorous selection process that prioritized high-quality study designs, such as randomized controlled trials (RCTs), which were included wherever possible to strengthen causal inferences. A systematic approach allowed for a comprehensive assessment of various lifestyle interventions on cardiac event-free survival in CAD patients. By including a diverse range of intervention types, such as intensive rehabilitation, diet modifications, exercise programs, and mobile health-guided approaches, the review provides a well-rounded view of how lifestyle modifications can be applied across different patient needs and settings. Heterogeneity was addressed narratively by comparing results across subgroups, such as intervention intensity, follow-up duration, and patient demographics, to provide context for observed variations in outcomes. However, the variability in patient adherence and intervention designs introduced challenges, as higher-intensity programs often demonstrated more pronounced effects, but their applicability in broader, real-world settings remains uncertain. Short follow-up durations in some studies further limited the ability to assess sustained long-term effects, necessitating caution when generalizing findings.

Publication bias was acknowledged as a potential limitation, though its assessment was limited due to the small number of included studies. A formal funnel plot analysis was not performed; however, studies reporting negative or null findings were included to reduce bias where available. These factors impacted the ability to draw definitive conclusions, particularly regarding the consistency of outcomes across different intervention intensities and settings. Future reviews could benefit from stricter inclusion criteria regarding follow-up duration and more standardized intervention protocols to improve comparability and robustness, alongside advanced methods for evaluating publication bias, such as Egger’s test or funnel plot analyses.

While this review highlights the positive impact of lifestyle interventions on cardiac outcomes in CAD patients, several research gaps remain. One key area is the need for more long-term studies, defined as those with follow-up durations of five years or more, to evaluate the sustainability of intervention benefits over extended periods. This duration is critical for assessing lasting impacts on cardiac event-free survival and reducing recurrent events, particularly in chronic conditions like CAD [23]. Methodologies like factorial designs could enable the evaluation of multiple intervention components, such as diet, exercise, and stress management, to identify which aspects are most beneficial [23]. Additionally, unique challenges faced by specific populations, such as frailty and limited mobility in older adults or post-surgical patients, warrant tailored approaches to improve outcomes. For instance, mHealth interventions like the Hartcoach program and telemedicine platforms offering remote monitoring and virtual coaching have shown promise in improving adherence and accessibility for high-risk groups [24]. Studies have also demonstrated the effectiveness of psychological and motivational support, such as cognitive-behavioral therapy (CBT) and motivational interviewing, in enhancing adherence and reducing anxiety, both of which are crucial for sustained lifestyle changes [24]. Addressing these areas could lead to more targeted, effective lifestyle programs that are adaptable to diverse patient populations and health settings.

## Conclusions

This systematic review affirms that lifestyle interventions play a crucial role in enhancing cardiac outcomes for patients with coronary artery disease (CAD), significantly improving long-term event-free survival and reducing recurrent cardiac events. Structured programs that incorporate dietary modifications, regular physical activity, stress management, and psychosocial support consistently demonstrate positive impacts on both physiological health markers and adherence to heart-healthy behaviors. These interventions, when complemented by standard pharmacological therapies, amplify benefits and provide a robust framework for secondary prevention. Among these, intensive programs and accessible approaches like mobile health and telephonic coaching stand out, particularly in improving adherence and outcomes across diverse patient populations. Clinicians and policymakers should integrate these interventions into routine CAD care, tailoring them to individual needs to maximize long-term benefits, reduce healthcare burdens, and improve overall quality of life.
